# Giant cell tumor of the anterior rib masquerading as a breast mass: a case report and review of current literature

**DOI:** 10.1186/1757-1626-3-51

**Published:** 2010-02-03

**Authors:** Nicole D Riddle, Hideko Yamauchi, Jamie T Caracciolo, David Cheong, Nazanin Khakpour, Marilyn M Bui

**Affiliations:** 1 Department of Sarcoma, Moffitt Cancer Center, Tampa, FL, USA; 2 Department of Radiology, Moffitt Cancer Center, Tampa, FL, USA; 3 Department of Anatomic Pathology, Moffitt Cancer Center, Tampa, FL, USA; 4 Department of Women's Oncology, Moffitt Cancer Center, Tampa, FL, USA; 5 Department of Pathology and Cell Biology, University of South Florida College of Medicine, Tampa, FL, USA

## Abstract

**Introduction:**

Giant cell tumor (GCT) is an aggressive, but usually benign bone neoplasm most commonly arising in the metaphysis/epiphyses of long bones. While they are categorized as benign tumors, they can be locally aggressive and clinically have metastatic potential. The most common locations of this tumor include the distal femur, proximal tibia, and distal radius. We report a GCT arising in an atypical location and mimicking a breast mass.

**Case Presentation:**

This case was diagnosed at a large cancer center in Florida. Pertinent clinical findings were obtained from chart review and inter-departmental consultation. Radiologically, the initial impression included a deep-seated breast cancer with local chest wall invasion. Further evaluation revealed the mass to be an expansile rib lesion with extraosseous soft tissue invasion. Histological examination of the biopsy specimen showed bland multi-nucleated giant cells and mononuclear cells whose nuclei were morphologically similar. No necrosis, pleomorphism or mitotic activity was identified. No chondroid or osseous elements were present.

**Conclusion:**

The histological features of bland mononuclear and multinucleated giant cells along with the lack of any additional mesenchymal elements led to the diagnosis of giant cell tumor. Resection of tumor was performed. The patient is disease free as of the last follow-up visit. This case is important as it shows where the physician must keep this diagnosis in mind whenever a deeply located breast mast is present.

## Introduction

Giant cell tumor (GCT) is an aggressive, but benign neoplasm most commonly arising in the metaphysis/epiphyses of long bones. It consists of multi-nucleated giant cells with surrounding spindle-shaped stromal cells. The majority of patients with GCT will present with a lytic geographic lesion that destroys the surrounding bone. Approximately 20% have an associated soft tissue mass with cortical breakthrough on radiographic images. Patient typically have a benign course, however, a remaining subset of patients (< 5%) will show evidence of metastatic involvement, usually to the lung. Local recurrence after surgical treatment can be as high as 50% [1]. Most commonly found in the long bones (distal femur, proximal tibia, and distal radius), we report an unusual case of GCT originating in the rib, an uncommon location for GCT.

## Case Report

**Figure 1:**
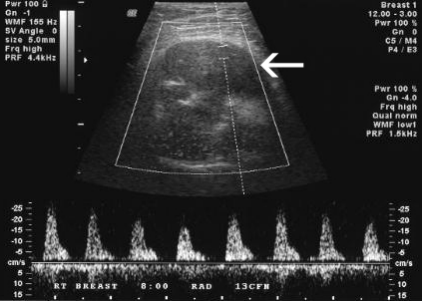
**Doppler breast ultrasound demonstrates hypo-echoic solid right breast mass with internal vascularity (arrow)**.

**Figure 2:**
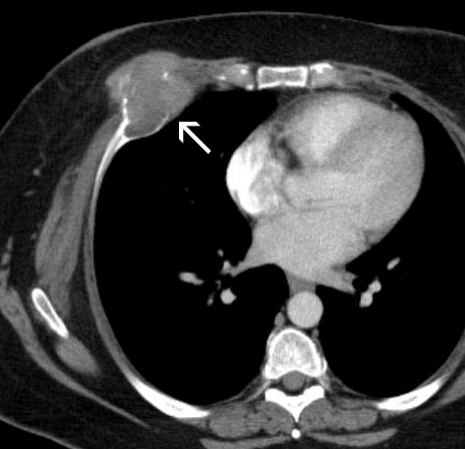
**Contrast-enhanced chest CT demonstrates a rounded anterior chest wall mass with osteolysis of the right fifth rib and a large soft tissue component**.

**Figure 3:**
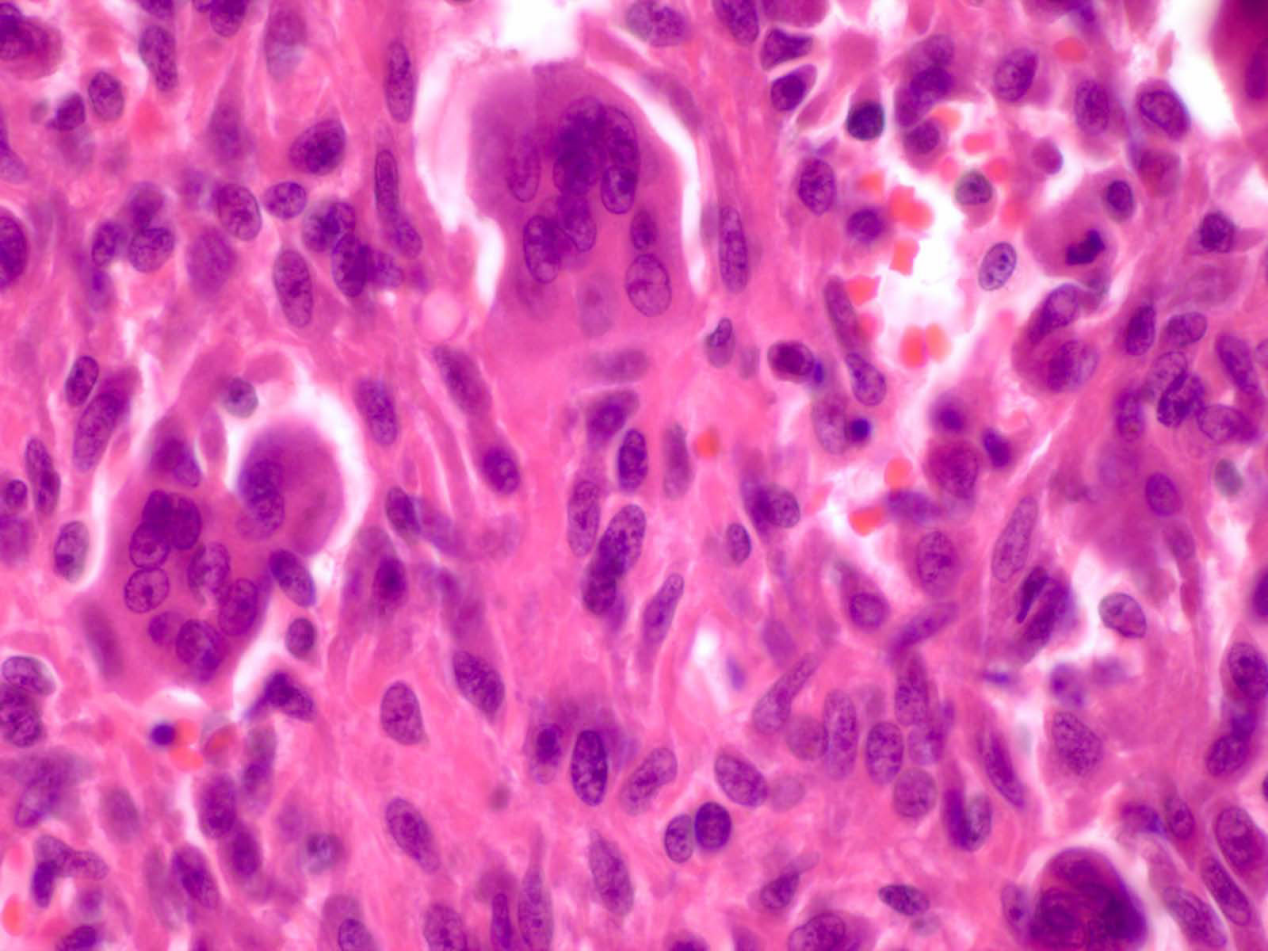
**Microscopically the tumor is composed of numerous multinucleated giant cells and uniform ovoid to spindle stromal mononuclear cells**. Note the nuclei of the stromal cells are similar to the giant cells, a typical feature of this tumor. (H&E stain, ×60).

A 37-year-old Caucasian female of European decent presented with a palpable mass of her right breast. She had no clinical symptoms and the overlying skin did not show any concerning changes. Her only complaint was for discomfort from the wire of her undergarments. She had a strong family history of breast cancer. Given the presentation of palpable breast mass, the initial imaging evaluation appropriately included mammography which demonstrated only dense breast parenchyma with no discrete breast mass or suspicious microcalcifications. A breast ultrasound revealed a 5.9 × 5.5 cm hypo-echoic, heterogeneous, solid mass with internal vascularity close to the chest wall (fig 1). A routine radiograph or chest x-ray was not indicated at initial presentation and was not performed. Subsequent contrast-enhanced chest computerized tomography (CT) demonstrated a rounded, expansile, lytic lesion involving the right fifth rib with cortical thinning and disruption, as well as local soft tissue invasion (fig 2). No lung lesions were identified. Given her family history, findings were suspicious for a breast tumor with local chest wall invasion. A fine needle aspiration (FNA) was performed and revealed scattered giant cells and was less than optimal for a definitive diagnosis. Follow-up incisional biopsy showed a hemorrhagic mass. Histologically, the tumor was composed of bland multi-nucleated giant cells and mononuclear cells (fig. 3). The final pathological diagnosis of the biopsy confirmed the diagnosis of a giant cell tumor. Following diagnosis, a wide resection of the lesion was performed including rib/chest wall/and tumor soft tissue extension. The effect on cosmesis was minimal as the rib resection occurred at the level of her natural breast fold, and included the prior surgical biopsy site. The parietal pleura were scarred to the overlying lesion. After dissection, the mass was successfully detached from the surrounding tissues. Macroscopic examination of the tumor showed an irregular, ill-defined, and rubbery mass measuring 5.0 × 5.0 × 4.5 cm. The central medullary portion of the rib was completely replaced by tumor. The cut surface of the tumor was variegated, pale-yellow to tan-pink, and focally hemorrhagic. Osseous and soft tissue surgical margins were free of tumor. The final pathological diagnosis was giant cell tumor of the right fifth rib. The patient remained well without evidence of recurrence at 1 year following surgery. Currently the only drawback is that she chooses not to wear underwire type support because of some scar sensitivity. She also does report some peri-incisional numbness, a sequelae of surgery to be expected with a re-resection in the dermatome distribution. No clinical photographs of before and after were obtained.

## Discussion

Giant cell tumor (GCT) of bone is an uncommon neoplasm, accounting for about 4-5% of all primary bone tumors. GCT are generally considered benign, but malignant cases can arise de-novo or via transformation from a benign neoplastic giant-cell lesion. The metaphyseal/epiphyseal zones of long bones are the most common sites, with 60% arising around the knee joint. Isolated cases have been reported in the scapula, sternum, patella, vertebra, skull, and talus [1]. Gupta and Mittal did a review of the English literature and discovered 15 cases involving the rib; however, most of those involved the posterior aspect and grew interiorly [2]. Due to its rarity, GCT arising from the chest wall is difficult to diagnose, especially when the tumor is located in the anterior arc of the ribs; the differential diagnosis easily lends itself to a primary breast lesion [3-9]. The case presented in this report stresses the difficulty in diagnosis and describes signs and symptoms that could be attributed to other conditions, such as breast cancer. GCT was not included in the initial differential diagnosis of the patient. The most likely initial diagnosis in this case was a deep-seated breast carcinoma.

Histopathologically, GCT is comprised of well-vascularized tissue of plump, spindled cells with numerous multinucleated cells. This may be difficult to differentiate from giant-cell rich osteosarcoma, malignant fibrous histiocytoma (MFH), chondroblastoma, aneurysmal bone cyst (ABC) and brown tumor. However, in GCT there is no significant nuclear pleomorphism, abnormal mitosis, atypical osteoid/chondroid matrix, nor is there associated hyperparathyroidism.

Treatment options include curettage with or without the use of phenol, alcohol, liquid nitrogen, methylmetacrylate, or bone graft and complete surgical resection of the affected segment of bone. Many of the currently accepted surgical treatment options have been associated with up to a 50% recurrence rate, which can be decreased with the use of adjuvant therapy [1,10]. Serum acid phosphatase levels have been suggested as a useful marker for diagnosis as well as for evaluation of the efficacy of treatment and the possibility of recurrence. The use of radiation therapy is not recommended in GCT due in part to reported association with malignant transformation of the lesions.

## Conclusion

The authors report a case of a GCT presenting as a palpable right chest wall mass in a woman with positive family history of breast cancer. Our case illustrates the fact that giant cell tumors of the anterior chest wall can be mistaken for breast masses. Fine needle or image guided core biopsy would be diagnostic if adequate specimen is obtained. The authors believe that the differential diagnosis of an anterior chest wall mass should include GCT of the ribs.

## Abreviations

ABC: Aneurysmal Bone Cyst; CT: Computerized Tomography; FNA: Fine Needle Aspiration; GCT: Giant Cell Tumor; MFH: Malignant Fibrous Histiocytoma.

## Consent

Informed consent was obtained from the patient for publication of this case report and accompanying images.

## Competing interests

The authors declare that they have no competing interests.

## Authors' contributions

NR, HY, and MB analyzed and interpreted the patient data regarding gross specimen and histopathology, including chart review, and were major contributors in writing the manuscript. JC performed the radiological examination of the lesion, and was a major contributor in writing the manuscript. DC contributed in consent the patient, obtaining information, analyzing the data, and editing the written report. NK contributed from the breast surgery point of view in the written report. All authors read and approved the final manuscript.
